# Mitochondrial Complex V Dysfunction in Neurodegeneration: Secondary Bystander or Primary Driver?

**DOI:** 10.3390/brainsci16080890

**Published:** 2026-08-20

**Authors:** Kate Erin Harris, Gerassimos Lascaratos, Kai-Yin Chau

**Affiliations:** 1 Department of Clinical and Movement Neurosciences, Queen Square Institute of Neurology, University College London, Royal Free Campus, London NW3 2PF, UK; kate.harris.25@ucl.ac.uk; 2 King’s College Hospital NHS Foundation Trust, Denmark Hill, London SE5 9RS, UK; 3 Section of Ophthalmology, School of Life Course Sciences, King’s College London, London SE1 7EH, UK

**Keywords:** Complex V, ATP synthase, mitochondria, bioenergetics, neurodegeneration, ATP synthesis, ATP hydrolysis

## Abstract

**Highlights:**

**What are the main findings?**

- Complex V abnormalities are reported across multiple neurodegenerative and neuro-ophthalmic disorders and are associated with impaired mitochondrial bioenergetics.
- Evidence for Complex V dysfunction varies considerably, with many studies relying on indirect bioenergetic measures rather than direct assessment of Complex V activity, assembly or directionality.

**What are the implications of the main findings?**

- Current evidence suggests that Complex V dysfunction is more likely a downstream consequence than a primary driver of disease.
- Complex V dysfunction is associated with impaired bioenergetics across multiple neurodegenerative and neuro-ophthalmic disorders, though the causal relationship remains unknown.
- Direct assessment of Complex V activity in disease-relevant human models is required to evaluate its therapeutic potential.

**Abstract:**

Background/Objectives: Mitochondrial Complex V (Complex V [CX-V], or ATP synthase) is the terminal enzyme of oxidative phosphorylation and is responsible for the majority of cellular ATP production. An increasing body of evidence suggests that CX-V dysfunction may contribute to mitochondrial impairment observed in neurodegenerative disease. This review evaluated current research on the structure, regulation, and function of CX-V, examined the consequences of CX-V dysfunction, and assessed its proposed role in neurodegenerative disorders. Methods: A comprehensive review of the published literature was carried out, with emphasis on primary research investigating CX-V structure and function, inherited CX-V disorders, and experimental evidence linking CX-V dysfunction to neurodegenerative disease. The reviewed studies used a range of experimental approaches, including structural biology, biochemical studies, patient-derived cellular models, animal models and post-mortem human tissue. Results: Current evidence demonstrates that disruption of CX-V impairs ATP production, alters mitochondrial membrane potential, and oxidative phosphorylation, and that pathogenic variants cause primary mitochondrial disease. Across Alzheimer’s disease, Parkinson’s disease, Huntington’s disease, amyotrophic lateral sclerosis/frontotemporal dementia, glaucoma and inherited optic neuropathies, alterations in CX-V activity, regulation and structural integrity are consistently associated with mitochondrial dysfunction. Direct evidence supporting CX-V as a primary driver of neurodegeneration remains very limited, with many observations originating from broader studies of general mitochondrial dysfunction. Conclusions: CX-V dysfunction represents a recurring feature of mitochondrial impairment across a variety of neurodegenerative disorders and may exacerbate neuronal vulnerability by disrupting cellular bioenergetics. Current evidence indicates that CX-V may serve as a common downstream target of multiple pathological pathways rather than acting as a primary pathological factor. Future studies require direct assessment of CX-V activity in clinically relevant human models and patient tissues to determine its contribution to disease progression and examine its potential as a therapeutic target.

## 1. Structure and Function of Complex V

**Figure 1:**
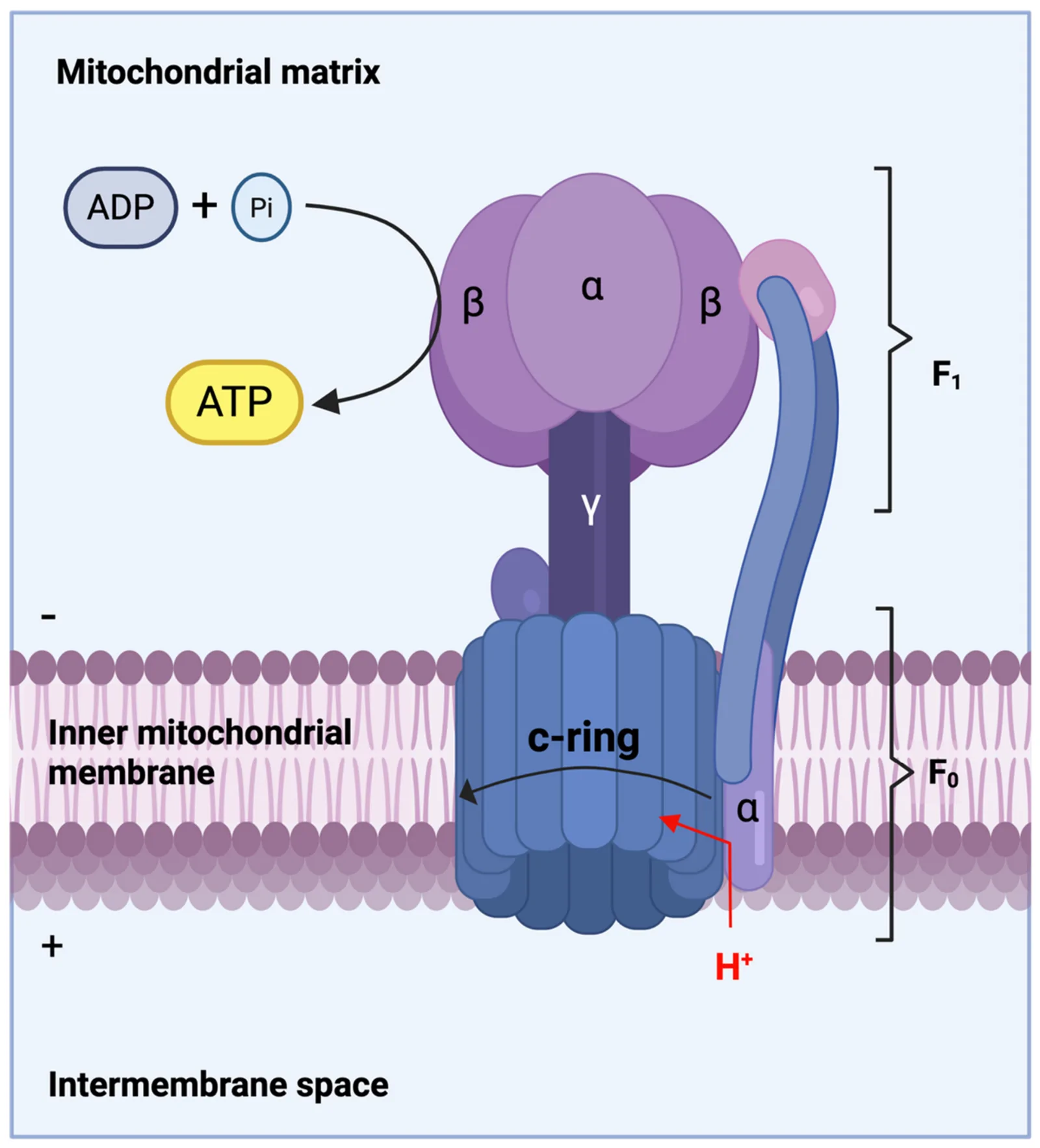
Simplified schematic of human mitochondrial Complex V (ATP synthase). Complex V is embedded within the inner mitochondrial membrane and comprises a membrane-embedded F_0_ motor and catalytic F_1_ domain. The F_1_ domain contains the catalytic α_3_β_3_ hexamer and central γ-subunit, while the F_0_ domain contains the c-ring and subunit a, with the F_1_ and F_0_ domains connected by the central and peripheral stalks. Protons (H^+^) flow down the proton gradient from the intermembrane space into the mitochondrial matrix through the F_0_ sector, driving rotation of the c-ring and central γ-subunit. This rotational energy is transmitted to the catalytic α_3_β_3_ hexamer of the F_1_ domain, where conformational changes within the β-subunits convert ADP and inorganic phosphate (Pi) into ATP. Created in BioRender. Harris, K. (2026) https://BioRender.com/kfdp8a0.

The pioneering work conducted by Professor John E. Walker laid the foundation for our current understanding of Mitochondrial Complex V (CX-V), more commonly known as ATP synthase [1]. CX-V is the terminal enzyme cluster of oxidative phosphorylation (OXPHOS) and is responsible for the majority of cellular ATP production. Located within the inner mitochondrial membrane, ATP synthase couples the protonmotive force (PMF) generated by the electron transport chain (ETC) to the synthesis of adenosine triphosphate (ATP) from adenosine diphosphate (ADP) and inorganic phosphate (Pi). The enzyme consists of two major domains: the membrane-embedded F_0_ sector and the catalytic F_1_ sector (see Figure 1). Together, these components enable the conversion of energy stored within a proton gradient into ATP.

### 1.1. Chemiosmotic Hypothesis and Proton Motive Force

As early as 1961, Mitchell proposed the chemiosmotic hypothesis, establishing that electron transport generates a proton motive force (PMF) across the inner mitochondrial membrane that subsequently drives ATP synthesis [2]. The PMF comprises both the mitochondrial membrane potential (ΔΨm) and proton concentration gradient (ΔpH). Experimental support was later provided by reconstitution of ATP synthase and bacteriorhodopsin within artificial phospholipid vesicles, demonstrating that an independently generated proton gradient was sufficient to drive ATP synthesis [3,4].

### 1.2. Rotary Mechanism

While Mitchell, Racker and Stoeckenius [3,4] established the source of energy driving ATP synthesis, the mechanism by which CX-V utilised this energy remained unknown. Walker [1] later described CX-V as a rotary molecular machine composed of two functionally distinct domains. The F_0_ domain is embedded within the inner mitochondrial membrane and is responsible for proton translocation, whereas the F_1_ domain projects into the mitochondrial matrix and contains the catalytic sites required for ATP synthesis. The coordinated activity of these domains allows the conversion of proton movement into catalytic activity, forming the basis of the rotary mechanism of CX-V.

### 1.3. Binding Change Mechanism

**Figure 2:**
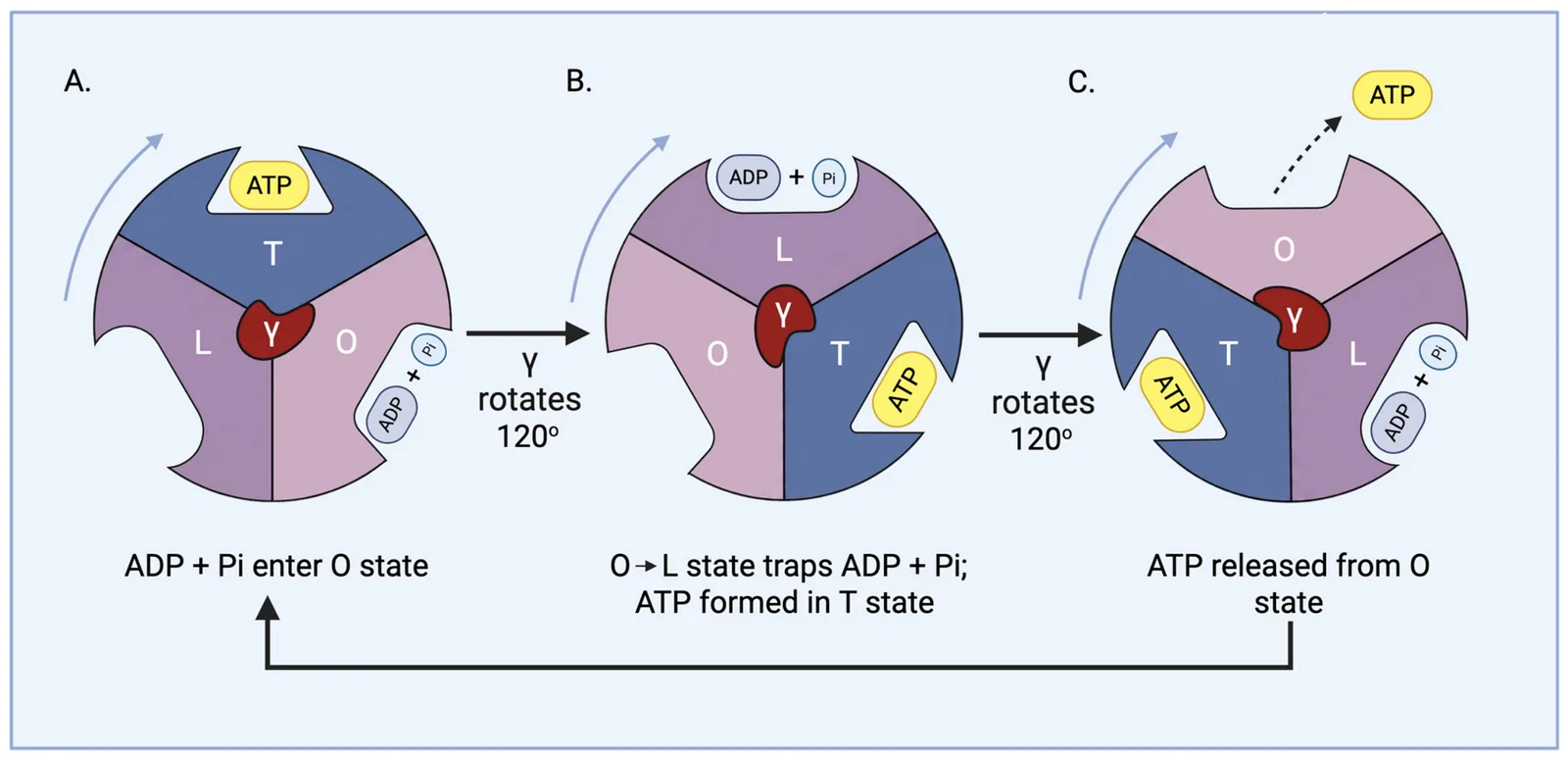
Boyer’s binding change mechanism in the F_1_ sector of human Complex V. The catalytic α_3_β_3_ hexamer contains three β-subunits that simultaneously adopt different conformational states: tight (T), loose (L) and open (O). (**A**) ADP and inorganic phosphate (Pi) enter the accessible open (O) state, while ATP remains tightly bound within the tight (T) catalytic site. (**B**) Proton-driven rotation of the central γ-subunit through 120° induces sequential conformational changes within each β-subunit. The substrate-containing O state transitions to the loose (L) state, trapping ADP and Pi within the catalytic site, while progression to the tight (T) state supports ATP synthesis and tight binding. The remaining catalytic sites progress through their respective conformations simultaneously. (**C**) A further 120° rotation converts the T state to the O state, allowing ATP to be released from the catalytic site, while the remaining β-subunits transition to the next stage of the catalytic cycle. Each complete 360° rotation of the γ-subunit drives three sequential catalytic events, producing one ATP molecule at each β-subunit as it cycles through the O, L, T conformational sequence. The energy derived from the PMF is required to drive these conformational transitions. Created in BioRender. Harris, K. (2026) https://BioRender.com/0ygfz7t.

The binding change mechanism dictates that the three catalytic β-subunits of the F_1_ domain cyclically transition through three asymmetrical conformational states: open (O), loose (L), and tight (T). Within this model, ADP and Pi first enter the open (O) state, where the catalytic site is accessible. Proton-driven rotation of the central γ-subunit then induces a conformational change to the loose (L) state, trapping ADP and Pi within the catalytic site. Further rotation promotes transition to the tight (T) state, where ATP is synthesised and tightly bound (see Figure 2). Therefore, energy derived from the PMF is not required for ATP formation itself, but rather for the conformational changes that enable ATP release from the catalytic site. This energy is transferred through proton translocation across the F_0_ motor, which drives rotation of the central γ-subunit and forces each β-subunit to cycle sequentially through the O, L and T conformations. This provided a mechanistic explanation for ATP synthesis that did not require a high-energy chemical intermediate [5].

Structural evidence supporting Boyer’s binding change mechanism was later provided by Abrahams and colleagues [6], who determined the crystal structure of bovine mitochondrial F_1_-ATPase at 2.8 Å resolution. The three catalytic β-subunits adopted different conformational states corresponding closely to the O, L and T states, providing structural evidence that distinct catalytic states coexist within a single ATP synthase complex. Subsequent structural studies have demonstrated that organisation of the F_1_ catalytic domain and its rotary mechanism are highly conserved in human CX-V [7].

### 1.4. Proton Flow: F_0_ to F_1_

While the crystal structure reported by Abrahams et al. provided structural support for Boyer’s binding change mechanism by revealing the three catalytic β-subunits in distinct conformational states, the mechanism linking these conformations to ATP synthesis remained unclear. A 2013 review by Professor Sir John Walker established that CX-V functions as a proton-driven rotary molecular machine composed of a membrane-embedded F_0_ motor and a catalytic F_1_ domain. Proton translocation through the F_0_ motor drives rotation of the c-ring and central stalk, inducing conformational changes within F_1_ that facilitate ATP synthesis and release [1].

More recent studies used molecular dynamics simulations to examine how proton transfer occurs within the F_0_ motor. It was proposed by Kubo and Takada [8] that protons enter and exit through two separate half-channels within the stationary α-subunit, while proton-binding amino acids on the rotating c-ring undergo repeated protonation and deprotonation to generate rotational movement. A highly conserved arginine residue within the α-subunit was suggested to maintain separation of the two half-channels, preventing direct proton diffusion across the membrane. Protons can therefore only cross the membrane by binding to and rotating the c-ring, ensuring that proton translocation remains tightly coupled to rotary motion and ATP synthesis.

These findings establish CX-V as a highly specialised energy-converting machine. CX-V is capable of transducing electrochemical energy into mechanical motion and finally into chemical energy in the form of ATP. Consequently, disruption at any stage of this process can impair cellular bioenergetics and contribute to disease [9].

## 2. Regulation of Complex V Activity

The primary role of CX-V is to synthesise ATP by utilising the PMF. CX-V, however, can operate in either the forward or reverse direction depending on ΔΨm and cellular energetic conditions. Regulation of CX-V activity is therefore crucial for maintaining cellular energy homeostasis, particularly during periods of metabolic stress, such as hypoxia or impaired electron transport, when ATP production becomes energetically unfavourable.

### 2.1. Role of IF1 in Inhibiting ATP Hydrolysis

ATPase inhibitory factor 1 (IF1), encoded by ATP5IF1, is a key regulator of CX-V activity [10]. Under normoxic conditions, CX-V uses the PMF generated by the ETC to synthesise ATP. Membrane depolarisation occurs in hypoxic or energetic-stress conditions; CX-V can operate in reverse and hydrolyse ATP to temporarily support ΔΨm. Sustained ATP hydrolysis can, however, accelerate ATP depletion during periods of energetic stress.

The role of IF1 in regulating CX-V activity during bioenergetic stress has been further clarified by Carroll et al. [11], who demonstrated that mammalian IF1 selectively inhibits ATP hydrolysis without significantly affecting ATP synthesis. This confirms its role as a unidirectional regulator of CX-V activity. The ability of IF1 to prevent wasteful ATP consumption while maintaining ATP production capacity implicates it as an important protective mechanism during mitochondrial stress.

Evidence suggests that IF1-mediated regulation extends beyond the inhibition of ATP hydrolysis. In *Saccharomyces cerevisiae*, deletion of the inhibitory peptides IF1 and STF1 (a nuclear-encoded regulatory peptide that works alongside IF1 to control CX-V activity) eliminated pH-dependent inhibition of ATP hydrolysis and increased susceptibility to mitochondrial uncoupling. These peptides stabilise the free F_1_-ATPase subcomplex, which can hydrolyse ATP but lacks regulation by the PMF. This suggests that IF1 influences CX-V function through mechanisms beyond simple catalytic inhibition [12].

These findings indicate that IF1 is more than a simple inhibitor of ATP hydrolysis; it functions as a dynamic regulator of cellular bioenergetics, maintaining ATP stores during periods of energetic stress and limiting excessive ATP consumption. This suggests that IF1 could represent a therapeutic target in diseases associated with mitochondrial dysfunction. However, despite growing mechanistic understanding of IF1-mediated regulation of ATP synthase, its role in neurodegenerative disease remains largely unclear.

### 2.2. ATP Synthesis vs. Hydrolysis

ATP synthase is primarily recognised for its role in ATP production, though the enzyme is inherently reversible. Under normal physiological conditions, the PMF generated by the ETC drives proton translocation through the F_0_ domain, powering rotation of the central stalk and promoting ATP synthesis within the F_1_ catalytic domain. However, when mitochondrial respiration is impaired and the PMF collapses, CX-V can switch direction and operate as an ATPase, hydrolysing ATP to pump protons across the inner mitochondrial membrane to preserve ΔΨm [13].

Experimental evidence suggests that this transition is more complex than a simple response to oxygen (O_2_) deprivation. Using osteosarcoma cells exposed to severe hypoxia (0.1% O_2_), Sgarbi and colleagues [14] demonstrate that CX-V retained the ability to synthesise ATP despite significantly reduced O_2_ availability. ΔΨm was assessed using tetramethylrhodamine methyl ester (TMRM) fluorescence, and ATP synthesis was quantified by luciferase-based bioluminescence assays. Despite severe hypoxia (0.1% O_2_), ΔΨm remained largely intact, and ATP synthesis continued at a lower rate than under normoxic conditions. This suggests that O_2_ deprivation alone is insufficient to induce ATP synthase reverse activity and instead implies that ATP hydrolysis occurs only after significant collapse of the PMF. In 143B osteosarcoma cells, FCCP-induced collapse of the PMF triggered ATP hydrolysis, enabling IF1-silenced cells to sustain ΔΨm more effectively than control cells, while accelerating cellular ATP depletion. The addition of oligomycin, an antibiotic that inhibits proton translocation in CX-V [15], eliminated this effect, confirming that CX-V ATP hydrolysis was responsible for sustaining ΔΨm. These findings suggest that CX-V only reverses direction after the collapse of the PMF, rather than in response to reduced O_2_ availability.

Carroll et al. [11] provided further mechanistic insight into how CX-V transitions between ATP synthesis and ATP hydrolysis. Using ATP synthesis and ATP hydrolysis assays in bovine submitochondrial particles, they demonstrated that IF1 selectively inhibits ATP hydrolysis while leaving ATP synthesis unaffected. These findings support the idea that ATP hydrolysis represents a tightly regulated adaptive response to bioenergetic stress, in which IF1 limits wasteful ATP consumption when ATP synthesis is no longer energetically favourable.

ATP hydrolysis can therefore transiently support ΔΨm following collapse of the PMF, although sustained reverse activity would progressively deplete cellular ATP. IF1 limits ATP hydrolysis under these conditions, preventing excessive ATP consumption. This allows ΔΨm to be maintained temporarily via reverse CX-V activity while limiting ATP depletion during prolonged energetic stress.

### 2.3. Impact of Mitochondrial Membrane Potential (ΔΨm)

The ΔΨm is a major determinant of CX-V directionality. As the primary electrical component of the PMF, ΔΨm provides the electrochemical energy required to drive ATP synthesis. When the PMF is sufficient, proton movement through the F_0_ domain drives enzyme rotation and promotes ATP production. Loss of ΔΨm, however, favours ATP hydrolysis, allowing CX-V to operate in reverse and pump protons across the inner mitochondrial membrane [11].

The relationship between ΔΨm and CX-V activity is bidirectional. While ATP synthesis depends on ΔΨm, ATP hydrolysis can help maintain ΔΨm when OXPHOS is compromised. Studies suggest that this reverse process may play an important role during bioenergetic stress. In models of respiratory chain dysfunction, inhibition of CX-V hydrolytic activity altered mitochondrial energetic responses, highlighting the contribution of reverse CX-V activity to cellular adaptation when ATP production is impaired [16].

These observations highlight that CX-V is highly responsive to changes in mitochondrial energetic state. Alterations in ΔΨm influence not only the efficiency of ATP production but also the direction of enzyme activity. Disruption of ΔΨ is a common feature of mitochondrial dysfunction; therefore, regulation of CX-V directionality under conditions of energetic stress may represent an important factor in cellular survival.

## 3. Functional Complexity of Complex V

### 3.1. Non-Binary Function

CX-V activity is continually influenced by the mitochondrial energetic state, with changes in PMF, ATP availability and regulatory proteins such as IF1 determining the direction of catalysis [1]. This allows CX-V to respond dynamically to changing energetic conditions.

The functional influence of CX-V may also extend beyond ATP production. Using extracellular flux analysis, mitochondrial enzyme activity assays and pharmacological inhibition with oligomycin, Du et al. [17] reported that sustained CX-V activity was required for maintenance of an OXPHOS-dependent anti-inflammatory macrophage phenotype. Inhibition of CX-V reduced the expression of programmed death-ligand 1 (PD-L1), an immune checkpoint protein that suppresses T-cell activation, and impaired the induction of regulatory T cells, linking mitochondrial CX-V activity to broader cellular programmes beyond bioenergetic homeostasis.

### 3.2. Intermediate Conformational States

ATP synthesis is driven by coordinated conformational changes within the three catalytic β-subunits of the F_1_ domain. While Boyer’s binding-change mechanism describes ATP synthesis as cycling between three catalytic states (O, L, and T), more recent cryo-electron microscopy (cryo-EM) studies suggest that these states are not discrete. Instead, CX-V appears to pass through multiple intermediate conformations as it rotates during catalysis.

Preservation of CX-V within intact *Polytomella* mitochondria captured the enzyme under physiological conditions, as revealed by cryo-electron tomography and subtomogram averaging. Structural classification identified six rotary positions of the central stalk and twenty-one substates within the F_1_ domain. These observations indicate that rotary catalysis progresses through multiple intermediate conformations rather than a small number of fixed structural states [7].

Similar mechanistic observations have been reported using the homologous archaeal Vacuolar (V/A)-ATPase. Although distinct from mitochondrial ATP synthase, its strong structural and functional conservation makes it a valuable model for investigating the mechanism of rotary catalysis. V/A-ATPase was reconstituted into proteoliposomes together with bacteriorhodopsin, allowing a PMF to be generated through light-driven proton transport. Following confirmation of ATP synthesis using luciferase-based assays, cryo-EM analysis identified multiple positions of the membrane rotor relative to the stator. Comparison of these structures revealed torsional deformation within both components during rotation, suggesting that conformational flexibility is a key feature of rotary catalysis rather than a consequence of structural instability [18].

### 3.3. Coordination of c-Ring Rotation and the Catalytic Cycle

Efficient ATP synthesis requires continuous coupling between proton translocation in F_0_ and catalytic events in F_1_. Although these processes are separated physically, disruption of their coordination would compromise energy transfer across the complex.

Structural analysis of V/A-ATPase under proton-driven synthesis conditions revealed a mismatch between the twelve-fold symmetry of the c-ring and the three catalytic sites of the V1 domain. Each proton translocation event produced approximately 30° of c-ring rotation, whereas ATP synthesis occurred in 120° catalytic steps. However, c-ring stoichiometry differs between species, and the twelve-fold symmetry observed in this V/A-ATPase should not be considered representative of mammalian mitochondrial CX-V. Mammalian mitochondrial ATP synthase contains a c_8_ ring, whereas yeast mitochondrial ATP synthase contains a c_10_ ring and larger c-rings are found in bacterial and chloroplast ATP synthases. In mammalian CX-V, translocation of eight protons drives one complete 360° rotation of the c_8_ ring, during which three ATP molecules are produced, corresponding to approximately 2.67 H^+^ per ATP [19]. The smaller c-ring therefore reduces the proton requirement for ATP synthesis compared with ATP synthases containing larger c-rings, increasing bioenergetic efficiency. Torsional strain in both the rotor shaft and the peripheral stalk was observed in Cryo-EM reconstructions, supporting a model in which elastic energy accumulates during successive rotational movements before contributing to larger catalytic transitions [7,18].

This relationship has also been examined computationally. Using all-atom free-energy simulations, Badocha et al. [20] investigated conformational changes occurring during the F_1_ synthesis cycle. Progression through the catalytic cycle was associated with coordinated closure of the ADP-binding β-subunit, coupled with opening of the ATP-releasing β-subunit during a 30–40° rotational substep of the γ-shaft. This indicated that energy associated with ADP binding could contribute directly to ATP release from a neighbouring catalytic site, supporting a highly coordinated mechanism throughout catalysis.

### 3.4. Complex V and the Mitochondrial Permeability Transition Pore

CX-V has also been linked to regulation of the mitochondrial permeability transition pore (mPTP), a high-conductance channel within the inner mitochondrial membrane that is induced by elevated matrix Ca^2+^ and oxidative stress. Prolonged mPTP opening causes loss of ΔΨm, impaired ATP synthesis, mitochondrial swelling and cell death, whereas transient opening may contribute to physiological Ca^2+^ release [21]. Cyclophilin D (CypD), an established regulator of mPTP sensitivity, binds to the OSCP subunit of the CX-V peripheral stalk, linking ATP synthase directly to permeability transition [21,22].

Several models have been used to explain how CX-V may contribute to pore formation. Purified ATP synthase dimers reconstituted into lipid bilayers have been shown to produce high-conductance channel activity with properties similar to the mPTP, supporting a dimer-associated model [22]. The F_0_ c-ring has also been proposed as a possible pore-forming component. Purified c-subunits form high-conductance channels, while sustained increases in matrix Ca^2+^ have been shown to enlarge the c-subunit ring and alter its association with F_1_. Reduced c-subunit expression also limits Ca^2+^ induced mitochondrial depolarisation and Ca^2+^ and ROS-associated cell death [23]. ATP synthase oligomers also contribute to the organisation of mitochondrial cristae, with apoptotic stimuli shown to disrupt these higher-order structures and alter cristae morphology [24].

However, the extent to which ATP synthase contributes directly to mPTP formation remains unclear. Genetic studies have shown that Ca^2+^ induced permeability transition can persist in the absence of assembled CX-V, with loss of ATP synthase increasing rather than preventing sensitivity to pore opening [25]. This suggests that CX-V is closely involved in regulating mPTP, although its exact role in pore formation remains uncertain.

Abnormalities affecting CX-V structure and regulation could therefore influence mPTP activity, thus providing a potential mechanism in which CX-V dysfunction may contribute to disturbances in ΔΨm, ATP production, Ca^2+^ homeostasis and oxidative stress. Dysregulation of these processes may further increase mitochondrial vulnerability and contribute to the cellular dysfunction associated with neurodegeneration.

## 4. Characterisation of Mitochondrial Dysfunction Caused by Variants of Genes Encoding Complex V

Human CX-V complex comprises 18 structural subunits encoded by 16 nuclear genes and two mitochondrial genes. Several nuclear-encoded proteins, including *ATPAF1*, *ATPAF2* and *TMEM70*, are required for the assembly, biogenesis and maintenance of the complex [26]. Pathogenic variants affecting CX-V provide a valuable opportunity to investigate the consequences of primary CX-V dysfunction. Unlike neurodegenerative disease, where CX-V abnormalities may arise secondary to broader cellular pathology, inherited CX-V disorders allow direct assessment of how disruption of individual subunits affects mitochondrial structure, bioenergetics and cellular function. Studies of both nuclear- and mitochondrial-encoded CX-V genes have consistently identified impaired OXPHOS, altered mitochondrial morphology and neurological dysfunction, highlighting the importance of CX-V in maintaining cellular energy homeostasis.

Genomic alterations affecting several of the CX-V genes listed in Table 1 have been associated with impaired complex activity and neurological disease. Pathogenic variants have been identified in nuclear-encoded genes, including *ATP5F1A*, *ATP5PO*, *ATP5F1B* and *ATP5MC3*, as well as the mitochondrial genes *MT-ATP6* and *MT-ATP8*. These variants have been associated with impaired CX-V activity and assembly, altered OXPHOS, reduced ATP production, and a range of neurological and neurodevelopmental phenotypes.

### 4.1. Nuclear Encoded Structural Subunits of Complex V

#### 4.1.1. *ATP5F1A*

Recent studies have provided important mechanistic insight into the consequences of *ATP5F1A* dysfunction. *ATP5F1A* encodes the α-subunit of the F_1_ catalytic domain, and pathogenic variants have been associated with both structural and functional mitochondrial abnormalities.

Across patient-derived cells and experimental models, *ATP5F1A* variants consistently impair CX-V function and mitochondrial bioenergetics. Lines et al. [29] described a recurrent de novo p.Arg207His variant in three unrelated neonates with failure to thrive, hyperlactataemia and hyperammonaemia. Patient-derived fibroblasts showed reduced CX-V activity and decreased α-subunit protein expression, while structural modelling suggested that substitution of Arg207 disrupted interactions between the α- and β-subunits adjacent to the catalytic site. Similar bioenergetic defects have been identified in fibroblasts carrying other heterozygous *ATP5F1A* missense variants, including reduced CX-V abundance, impaired OXPHOS and ATP production, mitochondrial depolarisation and respiratory uncoupling despite increased O_2_ consumption [30]. In zebrafish carrying the recurrent p.Gly418Arg variant, CX-V activity was reduced by approximately 30%, accompanied by disorganised mitochondrial cristae and motor dysfunction [31]. These findings indicate that impaired CX-V activity and mitochondrial bioenergetic dysfunction are common features of *ATP5F1A* variants across cellular and in vivo models.

Despite these shared mitochondrial abnormalities, evidence from different models suggests that the mechanisms underlying *ATP5F1A* dysfunction may vary between variants. Structural modelling of heterozygous missense variants identified in patient-derived cells showed clustering at interfaces between the α-, β- and γ-subunits of the F_1_ catalytic domain, where they were predicted to disrupt interactions required for ATP synthase function [30]. Expression of the human variants in *Caenorhabditis elegans* similarly induced mitochondrial stress and impaired CX-V activity, while increasing wild-type ATP5F1A expression partially ameliorated these defects, supporting a dominant-negative mechanism [30]. In contrast, partial rescue of the p.Gly418Arg zebrafish phenotype following expression of wild-type *ATP5F1A* was more consistent with loss of function. Overexpression of p.Gly418Arg in HEK293T cells also reduced α-subunit protein stability without altering mitochondrial localisation [31]. These findings suggest that *ATP5F1A* variants may disrupt CX-V through distinct molecular mechanisms despite producing similar bioenergetic defects.

The effects of *ATP5F1A* dysfunction also extend beyond ATP production. Zebrafish carrying p.Gly418Arg exhibited impaired motor neuron development, while transcriptomic analysis identified dysregulation of genes involved in neurotransmission and autophagy [31]. Clinical observations similarly demonstrate that the consequences of *ATP5F1A* dysfunction can be variable. Despite severe neonatal metabolic disturbance, all three individuals carrying p.Arg207His, as described by Lines et al., showed substantial clinical improvement during infancy [29]. This suggests that *ATP5F1A*-associated disorders may be mechanistically heterogeneous, despite sharing similar effects on mitochondrial function.

#### 4.1.2. *ATP5PO*

*ATP5PO* encodes the oligomycin sensitivity-conferring protein (OSCP), a component of the peripheral stalk that stabilises interactions between the F_1_ and F_0_ domains. Biallelic *ATP5PO* variants have been identified in individuals with severe neurodevelopmental and infantile mitochondrial disease, with patient-derived fibroblasts demonstrating impaired CX-V assembly and activity, mitochondrial respiratory dysfunction and reduced ATP production [32,33]. Although relatively few cases have been reported, these consistent biochemical abnormalities support *ATP5PO* disruption as a cause of primary CX-V deficiency.

#### 4.1.3. *ATP5F1B*

*ATP5F1B* encodes the β-subunit of the F_1_ catalytic domain. Heterozygous missense variants have been associated with early-onset isolated dystonia, with patient-derived fibroblasts showing severe and isolated reduction in CX-V activity despite largely preserved *ATP5F1B* expression, CX-V assembly and O_2_ consumption [34]. Structural modelling suggested disruption of catalytic F_1_ function rather than assembly of the mature complex. Although evidence remains limited, these findings identify *ATP5F1B* dysfunction as a potential cause of primary CX-V deficiency [34].

#### 4.1.4. *ATP5MC3*

*ATP5MC3* encodes subunit c of the F_0_ rotor, which contributes to the proton-conducting c_8_-ring. A homozygous *ATP5MC3* variant associated with childhood-onset developmental delay, spasticity and progressive neurological impairment resulted in reduced CX-V activity, impaired mitochondrial respiration and decreased ATP production in patient-derived fibroblasts. A Drosophila model similarly demonstrated impaired locomotor function and shortened lifespan, while expression of wild-type *ATP5MC3* restored mitochondrial function in vivo [35]. These findings demonstrate that disruption of the F_0_ rotor is sufficient to impair OXPHOS and produce neurological disease.

### 4.2. Mitochondrial DNA-Encoded Variants

#### 4.2.1. *MT-ATP6*

*MT-ATP6* encodes a core component of the F_0_ membrane domain that forms part of the proton translocation pathway within CX-V. Pathogenic variants in *MT-ATP6* are among the most frequently reported causes of primary CX-V dysfunction and have been associated with neurological disorders, including Leigh syndrome, neuropathy, ataxia and cognitive impairment [36,37]. Subsequent studies have expanded both the molecular and clinical spectrum of *MT-ATP6*-associated disease, demonstrating considerable variability in disease onset, severity and phenotype despite disruption of the same structural subunit [38,39,40].

Functional studies have established that pathogenic *MT-ATP6* variants impair OXPHOS by disrupting CX-V function. The novel m.8777T>C (p.Leu84Pro) variant was associated with reduced CX-V activity, impaired O_2_ consumption and decreased ATP production in patient-derived fibroblasts and cybrid cell lines. Homoplasmic cybrids exhibited marked bioenergetic impairment, while fibroblasts containing high levels of the variant demonstrated mitochondrial membrane hyperpolarisation, suggesting impaired proton transport across the inner mitochondrial membrane [41]. Truncating *MT-ATP6* variants have also been associated with isolated CX-V deficiency, increased ROS production and diverse neurological and multisystem phenotypes, reflecting the broad clinical spectrum of *MT-ATP6*-associated disease [42]. Evidence from multiple studies further indicates that pathogenic *MT-ATP6* variants disrupt proton translocation and ATP synthesis and, in some cases, impair CX-V stability or assembly depending on the underlying molecular defect [9].

Recently identified pathogenic *MT-ATP6* variants have further expanded the molecular spectrum of disease and were associated with impaired CX-V function and mitochondrial dysfunction [43]. Collectively, these findings demonstrate that pathogenic *MT-ATP6* variants consistently impair mitochondrial bioenergetics, reinforcing the essential role of *ATP6* in maintaining normal CX-V function.

#### 4.2.2. *MT-ATP8*

Compared with *MT-ATP6*, pathogenic variants affecting *MT-ATP8* have been less established, and their functional consequences remain less well characterised. *MT-ATP8* encodes a small subunit of the F_0_ membrane domain that is thought to contribute to CX-V stability.

Using in silico structural modelling and a Saccharomyces cerevisiae model, Panja et al. [44] demonstrated that the *MT-ATP8* p.Ile13Thr variant had minimal functional consequences in isolation, suggesting that some *MT-ATP8* variants may act primarily as modifiers of ATP synthase dysfunction rather than causing marked impairment independently. Despite preserved complex assembly, ATP synthesis and O_2_ consumption were reduced, suggesting that *MT-ATP8* variants can modify enzyme function through interaction with *MT-ATP6* rather than by disrupting assembly.

A novel pathogenic variant affecting the overlapping *MT-ATP6*/*MT-ATP8* region was identified in a child with isolated CX-V deficiency [45]. Although this finding supports the pathogenicity of variants involving the *ATP8* coding region, the variant affected both genes, preventing the specific contribution of *MT-ATP8* from being determined. There is limited available research to suggest that *MT-ATP8* dysfunction can contribute to primary CX-V deficiency alone.

The molecular heterogeneity associated with *MT-ATP6* and *MT-ATP8* variants is suggested by its broad clinical spectrum. In an international natural history study of 111 individuals with genetically confirmed *MT-ATP6* and *MT-ATP8* variants, neurological involvement was reported in 93% of patients, and muscle, ocular and cardiac manifestations were also common. Earlier disease onset was associated with greater morbidity, including metabolic acidosis, episodes of acute clinical deterioration and reduced survival, highlighting the substantial phenotypic variability associated with primary CX-V deficiency [46].

## 5. Evidence Linking Complex V to Neurodegenerative Disease

Neurodegenerative diseases comprise a heterogeneous group of neurological disorders characterised by progressive neuronal loss within the central or peripheral nervous system. Despite their diverse aetiologies and clinical manifestations, they share several interconnected pathological features, including altered energy homeostasis, which has been proposed as one of eight principal hallmarks of neurodegeneration [47]. Given the high energetic demands of neurons, mitochondrial dysfunction and impaired ATP production may contribute to neuronal vulnerability by disrupting energy-dependent processes. Mitochondrial dysfunction is, however, also observed in neurodegenerative diseases with predominantly non-mitochondrial aetiologies, and its origin frequently remains unknown [47]. Therefore, while genetic defects affecting CX-V provide direct evidence that ATP synthase dysfunction can cause neurological phenotypes [30,32,39], it has yet to be established whether CX-V dysfunction acts as a primary pathogenic driver in common neurodegenerative diseases. This uncertainty partly reflects the limited availability of specific approaches for assessing CX-V function and distinguishing ATP synthase from ATP hydrolase activity. CX-V dysfunction may instead, or additionally, contribute to altered energy homeostasis within a broader network of interconnected pathological processes that collectively influence neuronal vulnerability and degeneration.

Alterations in CX-V structure, activity and regulation have been reported across a range of neurodegenerative and neuro-ophthalmic disorders, including Alzheimer’s disease (AD), Parkinson’s disease (PD), Huntington’s disease (HD) and glaucoma. These findings are accompanied by broader bioenergetic abnormalities, including altered ATP production, ΔΨm, OXPHOS and mitochondrial integrity. However, these broader mitochondrial readouts are not specific measures of CX-V dysfunction and may reflect alterations in other components of the respiratory chain or mitochondrial network. This distinction is particularly important given the current limitations in specifically assessing CX-V function and directionality. Table 2 distinguishes direct evidence of CX-V dysfunction from indirect bioenergetic changes when evaluating whether CX-V contributes to neurodegenerative progression or instead represents a downstream consequence of broader mitochondrial stress.

### 5.1. Alzheimer’s Disease

AD is the most prevalent neurodegenerative disorder worldwide and is characterised by progressive cognitive decline associated with the accumulation of β-amyloid (Aβ) plaques and neurofibrillary tangles formed by hyperphosphorylated tau protein. Mitochondrial dysfunction and impaired OXPHOS are recognised features of AD pathology, with disruption of cellular bioenergetics contributing to neuronal dysfunction and cognitive decline [48]. Among the mitochondrial abnormalities reported in AD, increasing evidence suggests that CX-V may be a direct target of Aβ-mediated toxicity.

Reduced O-GlcNAcylation of the CX-V α-subunit (*ATP5A*), accompanied by decreased ATP synthase activity and reduced ATP levels, was demonstrated using post-mortem AD tissue, the 5×FAD mouse model, which overexpresses five familial AD mutations, and Aβ-treated cellular systems. Further biochemical analyses suggested that Aβ directly interacts with CX-V, disrupting the association between ATP5A and mitochondrial O-GlcNAc transferase (mOGT). Pharmacological restoration of O-GlcNAcylation partially rescued CX-V activity, indicating that CX-V dysfunction may represent a reversible component of AD-associated mitochondrial impairment [49]. These findings provided early evidence that Aβ can directly regulate CX-V, thus linking a defining pathological feature of AD to impaired mitochondrial bioenergetics.

This association was strengthened by identifying additional CX-V subunits targeted during disease progression. Expression of the oligomycin sensitivity-conferring protein (OSCP), a structural component of CX-V, was selectively reduced in both mild cognitive impairment and AD brain tissue. Aβ was shown to interact directly with OSCP, destabilising CX-V and reducing ATP production, alongside increased oxidative stress and increased susceptibility to mitochondrial permeability transition pore (mPTP) opening. Restoration of OSCP expression reduced several of these defects in both murine and human neuronal systems, suggesting that disruption of CX-V structural integrity may contribute directly to synaptic dysfunction and neuronal vulnerability [50].

The importance of OSCP was further highlighted by Gauba et al. [51], who identified Cyclophilin D (CypD) as an additional regulator of CX-V dysfunction in AD. Using post-mortem tissue, 5×FAD mice and CypD-deficient AD models, they demonstrated increased CypD–OSCP interactions during disease progression. These changes were associated with impaired CX-V activity, reduced mitochondrial respiration and ATP production. Genetic ablation of CypD preserved OSCP expression, restored mitochondrial bioenergetics and improved cognitive performance in 5×FAD mice [51]. This suggests that CypD-dependent destabilisation of OSCP exacerbates CX-V dysfunction and mitochondrial bioenergetic failure during AD progression. Further experiments showed overexpression of OSCP in 5×FAD mice reduced Aβ–OSCP interactions, restored CX-V regulation, preserved mitochondrial function and axonal mitochondrial transport, and attenuated synaptic loss, therefore improving spatial learning and memory abilities [52]. These findings suggest that preserving OSCP integrity protects mitochondrial function and reduces neuronal dysfunction in experimental models of AD [50,52].

Additional experimental models indicate that impaired CX-V activity is not restricted to a single AD model. Reduced CX-V activity, diminished ATP production and increased mitochondrial oxidative stress have been reported in APP-mutant neuronal models. Pharmacological interventions partially restored mitochondrial bioenergetics and improved CX-V activity [53,54]. Reduced F_0_F_1_ ATPase activity and ATP depletion have been demonstrated in the frontal cortex of 3×Tg-AD mice, further reinforcing impaired CX-V function as a feature of AD-associated mitochondrial dysfunction [55]. Although these studies primarily evaluated therapeutic interventions, each reported reduced CX-V activity and ATP production in untreated AD models, providing evidence that impaired CX-V function accompanies mitochondrial dysfunction across multiple experimental systems.

ATP synthase abnormalities have also been reported in ApoE4-expressing neuronal models. Proteomic analysis identified widespread alterations in mitochondrial proteins, including reduced abundance of multiple CX-V subunits, impaired respiratory capacity and diminished bioenergetic reserve, suggesting that alterations involving CX-V are not restricted to amyloid pathologies [56].

These studies identify CX-V as a target of AD-associated pathology. Although the mechanisms differ, the downstream consequences remain largely consistent: reduced CX-V activity, impaired ATP production, oxidative stress and mitochondrial dysfunction. Recent immunohistochemical analysis of human post-mortem tissue identified region-specific alterations in CX-V α-subunit and IF1 expression, particularly within the hippocampus, supporting the view that CX-V abnormalities are present in advanced stages of human disease [57]. A limitation, however, is that while current evidence largely demonstrates that AD-associated pathology can impair CX-V function, it remains unclear whether CX-V dysfunction drives neurodegeneration or is a downstream consequence of accumulating amyloid and other such pathological processes.

### 5.2. Parkinson’s Disease

PD is the second most common age-related neurodegenerative disorder and is characterised by progressive degeneration of dopaminergic neurons within the substantia nigra pars compacta, along with the accumulation of Lewy bodies containing α-synuclein. Mitochondrial dysfunction is considered a key feature of PD pathogenesis, and increasing evidence suggests that OXPHOS disruption contributes to neuronal loss [58]. Some studies suggest that several PD-associated proteins can directly interact with CX-V, impairing mitochondrial bioenergetics and increasing neuronal vulnerability.

Among these is α-synuclein, which has been implicated in the modulation of CX-V. Using super-resolution DNA-PAINT microscopy, proximity ligation assays and redox proteomics, Ludtmann et al. [59] demonstrated that oligomeric α-synuclein localises to CX-V in neuronal mitochondria. This association promoted the oxidation of the CX-V α-subunit and increased susceptibility to PTP opening, coupled with reduced ΔΨm. Oxidative modifications were identified within the CX-V β-subunit following exposure to α-synuclein oligomers, suggesting that CX-V may represent a direct target of pathogenic protein aggregation. Similar abnormalities were observed in human induced pluripotent stem cell (iPSC)-derived neurons carrying an SNCA triplication, a genetic cause of α-synuclein overexpression, supporting the physiological relevance of these findings. Overall, the study provided evidence that α-synuclein-induced mitochondrial dysfunction may, to some degree, be mediated through disruption of CX-V structural integrity and function.

The association between α-synuclein pathology and CX-V dysfunction has since been further solidified. Using limited proteolysis mass spectrometry and affinity purification mass spectrometry, Serdiuk et al. [60] identified structural alterations across multiple CX-V subunits following exposure to both monomeric and fibrillar α-synuclein. Fibrillar species produced distinct conformational changes compared with monomeric α-synuclein, suggesting that different aggregation states exert differential effects on CX-V structure and activity. These structural changes were accompanied by reduced ATP production in both isolated mitochondria and intact cells, supporting an association between α-synuclein-induced disruption of CX-V and impaired mitochondrial bioenergetics.

CX-V dysfunction has also been linked to alterations in DJ-1, a multifunctional protein encoded by the PD-associated gene PARK7. A direct interaction between DJ-1 and the CX-V β-subunit was demonstrated using co-immunoprecipitation and recombinant protein approaches. Loss of DJ-1 resulted in reduced CX-V activity, reduced cellular ATP levels, mitochondrial membrane depolarisation and increased inner membrane proton leakage. DJ-1 deficiency also disrupted CX-V stoichiometry through selective loss of the β-subunit, increasing exposure of the c-subunit associated with mitochondrial uncoupling. Consequently, impaired neuronal process outgrowth and reduced neuronal survival occurred, suggesting that DJ-1 contributes to mitochondrial homeostasis by maintaining CX-V assembly, coupling efficiency and bioenergetic function [61].

CX-V dysfunction has also been linked to mutations in coiled-coil-helix-coiled-coil-helix domain-containing protein 2 (CHCHD2), another PD-associated mitochondrial protein. Using MPP^+^-treated SH-SY5Y cells and an mPTP mouse model, Chen et al. [62] demonstrated that wild-type CHCHD2 promoted F_1_F_0_-ATPase assembly through direct interaction with the enzyme, compared to the pathogenic Thr61Ile mutation, which lost this ability. Expression of mutant CHCHD2 exacerbated mitochondrial dysfunction, behavioural deficits and dopaminergic neurodegeneration in vivo, suggesting that disruption of CX-V assembly may lead to PD-associated proteins impairing mitochondrial bioenergetics.

Studies using human brain tissue have observed DJ-1 interactions with CX-V in post-mortem PD brains and reported a significant reduction in DJ-1 association with CX-V in vulnerable substantia nigra *pars compacta* dopaminergic neurons. This interaction remained largely preserved in the relatively resistant ventral tegmental area. Reduced DJ-1-CX-V association occurred independently of overall mitochondrial DJ-1 abundance, indicating a targeted impairment in CX-V regulation rather than a general loss of the protein. This implies that abnormal regulation of CX-V-mediated proton leak may reduce mitochondrial efficiency and contribute to the selective neuronal vulnerability characteristic of PD [63]. Mitochondrial complex proteins, including *ATP5F1A*, have been studied within putative neuron-derived plasma exosomes as potential biomarkers of PD [64].

Despite mechanistic differences, the downstream consequences suggested in these studies remain consistent, showing impaired ATP production, altered ΔΨm and increased mitochondrial vulnerability observed across experimental systems. Human studies suggest that disruption of CX-V regulation is present within selectively vulnerable dopaminergic neurons. However, the relationship between CX-V and PD remains unclear, and additional research is needed to determine whether CX-V dysfunction contributes directly to disease progression or arises as a secondary effect of underlying pathology.

### 5.3. Huntington’s Disease

HD is an autosomal-dominant neurodegenerative disorder caused by the expansion of CAG repeats in the *HTT* gene, leading to the production of a mutant huntingtin protein containing toxic polyglutamine tracts. HD predominantly affects striatal medium spiny neurons and is characterised by early mitochondrial dysfunction, including impaired OXPHOS, mitochondrial fragmentation and disrupted calcium homeostasis [65]. Although much of the mitochondrial dysfunction reported in HD has been attributed to defects in Complexes II and III, some studies suggest that CX-V dysfunction may also contribute to the bioenergetic deficits leading to neuronal degeneration.

Evidence implicating CX-V dysfunction in HD has been reported in studies using patient-derived iPSCs, neural stem cells and CRISPR-corrected isogenic controls. Metabolic analyses revealed reduced OXPHOS, lower ATP-linked respiration and reduced spare respiratory capacity in HD cells, indicating substantial impairment of mitochondrial energy production. Assessment of ΔΨm following oligomycin treatment showed greater depolarisation in HD cells than in controls. These findings are suggestive of increased reverse ATPase activity, in which ATP hydrolysis may contribute to the maintenance of ΔΨm rather than ATP synthesis. The ATP/ADP ratio and complex III activity further supported the presence of impaired cellular bioenergetics, although these measurements are indirect and do not specifically demonstrate ATP hydrolysis by CX-V. Unlike the structural disruption of CX-V observed in AD and PD, these findings are consistent with the possibility that ATP synthase remains functionally active but operates in reverse under conditions of bioenergetic stress. Although this may initially represent a compensatory response to preserve ΔΨm, sustained ATP hydrolysis is likely to accelerate cellular ATP depletion, potentially exacerbating neuronal dysfunction and degeneration. Correction of the mutant *HTT* allele rescued many of these abnormalities [66].

While studies in AD and PD have largely focused on structural disruption of CX-V or impaired ATP production, the research presented for HD shows that disease-associated mitochondrial dysfunction may alter the directionality of CX-V activity itself. Neuronal survival may be compromised if increased reverse CX-V activity leads to greater consumption of cellular ATP reserves to sustain ΔΨm, potentially worsening cellular energy deficits under pathological conditions.

Using a transgenic minipig model, reduced abundance of the CX-V OSCP was observed, along with OXPHOS impairment and altered mitochondrial structure. Reduced OSCP expression may contribute to mitochondrial dysfunction by altering the regulation of the mPTP [67]. In primary striatal astrocytes isolated from the R6/1 transgenic mouse model of HD, increased ATP-linked respiration, mitochondrial oxidative stress and disrupted mitochondrial dynamics were reported, consistent with a hypermetabolic phenotype [68]. However, CX-V was not directly investigated.

CX-V dysfunction may contribute to the bioenergetic abnormalities observed in HD. However, compared with AD and PD, direct evidence remains very limited. Much of the available literature focuses on broader mitochondrial dysfunction rather than CX-V specifically. Whether alterations in CX-V represent a primary pathogenic mechanism or arise as a downstream consequence remains unresolved. Further studies directly investigating CX-V in neuronal models of HD will be required to determine its contribution to disease progression.

### 5.4. Frontotemporal Dementia and Amyotrophic Lateral Sclerosis (ALS/FTD)

FTD is characterised by progressive degeneration of the frontal and temporal lobes, resulting in behavioural, executive and language impairment. ALS is characterised by degeneration of upper and lower motor neurons, leading to progressive muscle weakness. Despite being clinically distinct, the two diseases exist along a disease spectrum and share overlapping genetic and pathological mechanisms, with approximately 15–20% of patients with ALS also exhibiting the behavioural variant of FTD [69]. The chromosome 9 open reading frame 72 (*C9ORF72*) hexanucleotide repeat expansion (GGGGCC) is the most common genetic cause of ALS and a major genetic cause of FTD, and has been associated with mitochondrial dysfunction and impaired cellular bioenergetics [70].

Using an inducible poly(GR)-expressing mouse model, primary cortical neurons and post-mortem tissue from individuals with *C9ORF72*-associated ALS/FTD were analysed. It was demonstrated that the arginine-rich dipeptide repeat protein poly(GR) binds the CX-V α-subunit (*ATP5A1*), promoting its ubiquitination and proteasomal degradation. This was associated with reduced CX-V activity, impaired ATP production, and mitochondrial morphological abnormalities. Restoration of *ATP5A1* expression rescued CX-V activity, improved mitochondrial morphology and increased neuronal survival. Reduced *ATP5A1* abundance was observed in post-mortem *C9ORF72* tissue, supporting the clinical relevance of these findings [70].

It remains unclear to what extent CX-V dysfunction contributes to disease progression in vivo. Despite *ATP5A1* degradation being identified as an early consequence of poly(GR) toxicity, establishing whether CX-V dysfunction represents a common pathogenic mechanism across the ALS/FTD spectrum or is predominantly restricted to *C9ORF72*-associated disease remains to be established.

### 5.5. Glaucoma

Glaucoma comprises a heterogeneous group of neurodegenerative disorders characterised by progressive loss of retinal ganglion cells (RGCs) and optic nerve damage, ultimately leading to irreversible visual impairment and in some cases blindness [71]. Mitochondrial dysfunction is increasingly associated with disease pathogenesis, disturbances in OXPHOS and ATP homeostasis being key features.

Transcriptomic profiling of primary rat RGC cultures and in vivo endothelin-1 (ET-1)-induced glaucoma models identified OXPHOS as the most significantly disrupted mitochondrial pathway following ET-1 exposure. Several mitochondrial proteins, including the CX-V F_0_ subunit *ATP5H*, showed reduced expression, accompanied by reduced mitochondrial ATP production. These findings support the presence of impaired mitochondrial bioenergetics during ET-1-mediated RGC degeneration and suggest that altered CX-V subunit expression may accompany glaucomatous pathology [72].

Fibroblasts derived from patients carrying the E50K OPTN mutation exhibited fragmented mitochondrial networks, disrupted cristae architecture, increased O_2_ consumption and elevated proton leak despite impaired OXPHOS. Functional analyses demonstrated increased activity of the CX-V c-subunit leak channel, promoting ATP hydrolysis. Treatment with an ATP synthase-modulating compound, dexpramipexole, restored forward CX-V activity, improved mitochondrial ultrastructure and normalised cellular metabolism, further implicating CX-V dysfunction in the observed bioenergetic abnormalities [73,74].

Sequencing of mitochondrial genes involved in cellular energy metabolism identified several glaucoma-associated variants, including *MT-ATP6*. The m.8860A>G variant was associated with glaucoma-related endophenotypes, particularly alterations in the vertical cup-to-disc ratio. However, the CX-V function was not experimentally assessed, and as such, the biological consequences of these variants remain uncertain [75].

Altered ATP metabolism has also been identified in clinical samples. Metabolomic analysis of aqueous humour from patients with primary open-angle glaucoma revealed elevated ATP levels, accompanied by changes in inflammatory cytokines and metabolic pathways linked to cellular stress. Rather than reflecting elevated cellular bioenergetics, an increase in extracellular ATP may promote microglial activation and metabolic remodelling through purinergic signalling pathways [76]. Although CX-V activity was not directly examined, these findings suggest that dysregulated ATP signalling may contribute to disease progression.

Alterations associated with CX-V have therefore been identified alongside impaired mitochondrial bioenergetics, altered ATP metabolism and disrupted regulation of CX-V activity in glaucoma models. However, the available evidence predominantly demonstrates associations, and the causal relationship between CX-V dysfunction and glaucoma remains unresolved.

### 5.6. Other Optic Neuropathies

CX-V dysfunction may also contribute to inherited optic neuropathies characterised by RGC degeneration. In dominant optic atrophy (DOA), RGCs differentiated from patient-derived iPSCs carrying the *OPA1* R445H variant displayed disrupted mitochondrial networks, abnormal cristae architecture, reduced ΔΨm and increased susceptibility to mPTP opening. Impaired maintenance of ΔΨm following oligomycin treatment suggested increased reliance on reverse CX-V activity (hydrolysis) to sustain ΔΨm, suggesting that altered CX-V function may contribute to disease pathogenesis [77]. In Leber’s hereditary optic neuropathy (LHON), mutations in the X-linked mitochondrial protein *PRICKLE3* impaired CX-V assembly, stability and activity through direct interaction with *ATP8*, reducing ATP production and promoting RGC degeneration. These abnormalities were exacerbated in the presence of the pathogenic *MT-ND4* mutation, highlighting functional interactions between Complexes I and V during disease progression [78].

Further evidence implicating CX-V in inherited optic neuropathies comes from rare pathogenic *MT-ATP6* variants. One patient carrying the m.8969G>A MT-ATP6 variant presented with a classical LHON-like phenotype. This was characterised by subacute bilateral visual loss and optic atrophy, further extending the clinical spectrum of CX-V disorders [79]. However, this clinical association does not establish a direct mechanistic role for CX-V dysfunction in RGC degeneration. Despite their distinct genetic causes, both DOA and LHON show evidence of altered CX-V function, suggesting that CX-V dysfunction may contribute to RGC vulnerability across inherited optic neuropathies.

### 5.7. Mechanistic Links Between Complex V Dysfunction and Neurodegeneration

Across neurodegenerative disorders, several mechanisms may link CX-V dysfunction to neuronal damage [47]. Reduced CX-V activity or impaired assembly can limit ATP production and compromise cellular bioenergetics, which may be particularly important in neurons because of their high energy requirements. Alterations in CX-V can also affect ΔΨm, proton conductance and mitochondrial coupling, with subsequent effects on oxidative stress and mitochondrial function. Changes involving OSCP and other CX-V components may additionally influence susceptibility to mPTP opening, providing a potential link between impaired bioenergetics and mitochondrial dysfunction during cellular stress. In some disease models, reverse CX-V activity may temporarily maintain ΔΨm through ATP hydrolysis, although prolonged reverse activity can further deplete cellular ATP. Together, these mechanisms suggest that CX-V dysfunction may contribute to neurodegeneration through several interconnected effects rather than through impaired ATP synthesis alone [47].

## 6. Critical Appraisal and Future Directions

### 6.1. Current Evidence Base

This review aimed to identify literature implicating CX-V dysfunction across a range of neurodegenerative and optic neurodegenerative disorders. Collectively, these studies suggest that disturbances in CX-V activity, regulation and structural integrity may contribute to impaired mitochondrial bioenergetics and neuronal vulnerability. However, despite the growing number of publications referring to CX-V, more often referred to as ATP synthase, direct evidence remains limited. CX-V was often discussed as one component of broader mitochondrial dysfunction rather than serving as the primary focus of investigation. Consequently, conclusions regarding CX-V involvement were frequently inferred from alterations in ATP production, ΔΨm or respiratory function, rather than from direct assessment of CX-V activity or regulation.

One of the challenges encountered during this review was the lack of disease-specific studies centred around CX-V. Although numerous publications reported mitochondrial dysfunction, comparatively few have determined the specific contribution of CX-V dysfunction to disease pathogenesis. Therefore, the disease spectrum considered within this review was intentionally broadened to include additional neurodegenerative and optic neuropathic disorders. This enabled identification of recurring themes across diverse pathologies, such as adjustments to ΔΨm and ATP synthase structure, while highlighting the limited depth of evidence currently available within individual diseases. This broader perspective, therefore, reflects a key limitation within the field, rather than a shortcoming of the review.

### 6.2. Experimental Heterogeneity

A further challenge encountered during this review was the considerable experimental heterogeneity across the available literature. Studies investigating CX-V utilised a diverse range of experimental systems, including yeast, purified mitochondria, immortalised cell lines, patient-derived fibroblasts, iPSC-derived neurons, primary neuronal cultures, animal models and post-mortem human tissue. Whilst this diversity has advanced understanding of CX-V biology, each model addresses distinct biological questions and possesses inherent strengths and limitations. Mechanistic studies performed in simplified experimental systems often provide detailed insight into CX-V structure and regulation but lack disease-specific physiological relevance. Patient-derived models and post-mortem tissue more accurately reflect human pathology but are frequently limited by sample availability, disease heterogeneity and the inability to distinguish primary pathogenic mechanisms from downstream consequences.

One key observation throughout this review was that relatively few studies placed CX-V at the centre of their investigations. Although CX-V was frequently implicated within broader discussions of mitochondrial dysfunction, it was rarely studied directly. Consequently, supporting evidence for many mechanistic observations remained limited despite a substantial body of literature describing mitochondrial dysfunction. This made direct comparison between studies challenging, as investigations often examined different aspects of CX-V biology using different experimental approaches and disease models. Establishing consistent mechanistic themes frequently relied on integrating complementary findings rather than direct replication. Improving methodological consistency, alongside the development of more robust and accessible approaches for directly assessing CX-V function and regulation, would greatly strengthen future investigations and allow for more meaningful comparisons across studies.

### 6.3. Is CX-V a Driver or a Consequence?

A key question emerging from this review is whether CX-V dysfunction acts as a primary driver of disease or instead occurs as a downstream consequence of other pathological processes. Although definitive conclusions cannot yet be drawn, the available evidence suggests that, in most cases, CX-V dysfunction is more likely to occur as a downstream consequence of disease than as the initiating pathological event. Across the diseases discussed in this review, the initiating pathological processes differ substantially, ranging from β-amyloid accumulation and α-synuclein aggregation to mutant *HTT* expression and inherited mitochondrial defects. Despite these differences, disruption of CX-V consistently corresponds with impaired ATP production, altered ΔΨm, structural abnormalities and reduced mitochondrial integrity.

This pattern suggests that CX-V dysfunction may represent a common downstream response to diverse pathogenic factors rather than a primary driver of pathology. However, this interpretation is challenged by studies demonstrating that restoration of CX-V integrity can improve disease-associated phenotypes. For example, overexpression of OSCP restored CX-V integrity, improved mitochondrial function and ameliorated cognitive deficits in an AD mouse model [52]. Although these findings do not demonstrate that CX-V dysfunction initiates disease, they suggest that, once established, it can become an active contributor to disease progression by amplifying bioenergetic failure. This, however, should not be interpreted as evidence that CX-V plays only a passive role in disease progression. Once CX-V function becomes compromised, the resulting reductions in ATP availability, altered maintenance of ΔΨm and impaired OXPHOS are likely to exacerbate mitochondrial dysfunction within cells already experiencing considerable metabolic stress. Given the high energetic demands of neurons, even modest impairments in ATP synthesis may reduce cellular resilience and accelerate neurodegenerative progression.

Current evidence suggests that CX-V dysfunction is rarely the initiating pathological event in neurodegenerative disease but instead represents a common point of convergence across diverse pathogenic processes. However, once established, impaired ATP synthesis, altered ΔΨm and disrupted mitochondrial bioenergetics are likely to accelerate neuronal dysfunction and disease progression. Determining whether modulation of CX-V can alter disease progression, rather than simply correct downstream bioenergetic abnormalities, therefore remains an important objective for future research.

### 6.4. Future Research

Future research should aim to move towards more direct investigations of CX-V function, rather than the broader, indirect approach often seen in current studies. ATP production, O_2_ consumption and ΔΨm were frequently used as indirect indicators of CX-V activity, despite providing only indirect evidence of alterations in CX-V activity or regulation. Robust, reproducible and accessible methods for directly assessing CX-V function within disease-relevant models will therefore be essential for establishing its contribution to disease pathogenesis.

Another aim for future research would be to distinguish between impaired ATP synthesis and increased ATP hydrolysis. Few studies directly assessed the directionality of CX-V activity despite the potential implications for neuronal survival and disease progression. Longitudinal investigations using patient-derived neuronal models and disease-relevant tissues will be necessary to determine whether CX-V dysfunction occurs before irreversible neuronal damage or arises as a result of established pathology.

Finally, the identification of reliable biomarkers of CX-V dysfunction would facilitate earlier detection of mitochondrial impairment and improve assessment of therapeutic efficacy. Future therapeutic strategies should also consider targeting CX-V regulation, assembly and stability, rather than focusing on restoring global mitochondrial function. These strategies could help determine whether modulating CX-V influences disease progression or solely alleviates the downstream effects of mitochondrial dysfunction.

### 6.5. Conclusions

Current evidence suggests that CX-V dysfunction is more likely to arise as a downstream consequence of diverse pathogenic mechanisms than as a primary pathological event. Its recurrent involvement suggests that CX-V is a potential point of mechanistic convergence in mitochondrial dysfunction. While the initiating pathological processes differ substantially between diseases, many ultimately converge on impaired ATP production, altered ΔΨm and reduced mitochondrial integrity, highlighting the relevance of CX-V in disease progression.

Progress in this area will depend on improving access to approaches that can directly assess CX-V activity and regulation, particularly in disease-relevant human models. These advances will be important for addressing the uncertainties highlighted throughout this review and for determining whether CX-V represents a viable therapeutic target capable of slowing neurodegenerative progression. Overall, CX-V should be considered not simply as another component of OXPHOS, but as a promising and increasingly important focus for future neurodegenerative research.

## Figures and Tables

**Table 1:** Structural subunits of the human mitochondrial Complex V. Genes and their proteins are categorised by genomic origin (nuclear-encoded or mitochondrial DNA-encoded) and grouped according to their location within the enzyme complex, including the F_1_ catalytic domain, central stalk, peripheral stalk, F_0_ rotor (c_8_-ring), and F_0_ membrane domain. The mammalian F_0_ rotor contains eight c-subunits forming the c_8_-ring, while mitochondrially encoded *MT-ATP6* (subunit a) forms the proton-translocation interface with the c_8_-ring [26,27,28].

Encoded by	ATP Synthase Region	Gene	Protein (Subunit)
Nuclear	F_1_ catalytic domain	*ATP5F1A*	α
		*ATP5F1B*	β
	Central stalk	*ATP5F1D*	δ
		*ATP5F1E*	ε
		*ATP5F1G*	γ
	Peripheral stalk	*ATP5PO*	OSCP
		*ATP5PB*	b
		*ATP5PD*	d
		*ATP5PF*	F6
	F_0_ rotor (c_8_-ring)	*ATP5MC1*	c
		*ATP5MC2*	c
		*ATP5MC3*	c
	F_0_ membrane domain	*ATP5ME*	e
		*ATP5MF*	f
		*ATP5MG*	g
		*ATP5MD*	DAPIT
Mitochondria		*MT-ATP6*	a
		*MT-ATP8*	A6L

**Table 2:** Summary of reported Complex V alterations across neurodegenerative disorders and their possible consequences. Reported changes in CX-V structure, activity and regulation are shown alongside their effects on mitochondrial function and the possible mechanisms through which they may contribute to neuronal dysfunction. The evidence varies between disorders, and these associations do not necessarily indicate that CX-V dysfunction is a primary cause of disease.

Neurodegenerative Disorder	Experimental Model	CX-V Alterations	Functional Consequence	Possible Mechanism Linking CX-V Dysfunction to Neurodegeneration	Direct or Indirect CX-V Readout
Alzheimer’s disease (AD)	Post-mortem human brain 5×FAD and 3×Tg-AD mice Aβ-treated cellular models APP-mutant neuronal models ApoE4-expressing neurons	Reduced α-subunit O-GlcNAcylation Aβ interaction with CX-V and OSCP Reduced OSCP expression Increased CypD–OSCP interaction Reduced abundance of multiple CX-V subunits	Reduced CX-V activity and ATP production Impaired respiration and bioenergetic reserve Increased oxidative stress and susceptibility to mPTP opening	Aβ-associated disruption of CX-V regulation and structural integrity may impair ATP synthesis and increase oxidative and mPTP-associated mitochondrial stress, contributing to synaptic dysfunction and neuronal vulnerability	Mixed (largely direct): CX-V activity, subunit abundance, protein interactions and ATP synthase regulation measured directly Broader bioenergetic readouts also included
Parkinson’s disease (PD)	Neuronal mitochondria α-synuclein-treated cells/isolated mitochondria SNCA-triplication iPSC-derived neurons DJ-1-deficient models MPP^+^-treated SH-SY5Y cells Mouse models Post-mortem human brain	α-Synuclein association with and oxidative modification of CX-V Conformational changes in CX-V subunits Reduced DJ-1–CX-V interaction Altered CX-V stoichiometry Impaired CHCHD2-mediated CX-V assembly	Reduced ATP production and CX-V activity ΔΨm loss Increased proton leak Altered coupling Increased susceptibility to PTP opening	Pathogenic α-synuclein and PD-associated proteins may disrupt CX-V structure, assembly and regulation, reducing mitochondrial efficiency and increasing vulnerability of dopaminergic neurons	Direct: CX-V interactions, structural changes, assembly, activity and subunit alterations examined directly
Huntington’s disease (HD)	Patient-derived iPSCs and neural stem cell CRISPR-corrected isogenic controls Transgenic minipig R6/1 mouse-derived striatal astrocytes	Evidence of increased reverse CX-V activity Reduced OSCP abundance in a transgenic model Broader OXPHOS dysfunction	Reduced ATP-linked respiration and spare respiratory capacity ATP hydrolysis to sustain ΔΨm Mitochondrial oxidative stress and altered dynamics	Reverse CX-V activity may temporarily preserve ΔΨm during energetic stress but progressively consume cellular ATP reserves. Reduced OSCP may additionally alter CX-V/mPTP regulation; however, direct CX-V evidence remains limited	Mixed (largely indirect): Oligomycin responses and ΔΨm support altered CX-V directionality OSCP abundance measured directly Several studies assess broader mitochondrial function without direct CX-V measurement
ALS/Frontotemporal dementia (ALS/FTD)	Inducible poly(GR) mouse model Primary cortical neurons Post-mortem C9ORF72-associated ALS/FTD tissue	C9ORF72-associated poly(GR) binding to the CX-V α-subunit, promoting ubiquitination and degradation	Reduced CX-V activity and ATP production Altered mitochondrial morphology	Loss of the CX-V α-subunit may directly impair ATP synthase function and mitochondrial bioenergetics, contributing to neuronal vulnerability in C9ORF72-associated ALS/FTD	Direct: CX-V α-subunit interaction, abundance and activity measured directly
Glaucoma	Primary rat RGC cultures ET-1-induced rat glaucoma model E50K OPTN patient fibroblasts Human genetic studies Aqueous humour metabolomics	Reduced expression of a CX-V F_0_ subunit Increased c-subunit leak-channel activity MT-ATP6 variants associated with glaucoma-related phenotypes	Reduced mitochondrial ATP production Increased proton leak and ATP hydrolysis Impaired OXPHOS and altered ATP metabolism	Altered CX-V expression and proton conductance may reduce mitochondrial efficiency and ATP availability in RGCs. Current evidence predominantly supports association rather than a direct causal role	Mixed: Direct CX-V expression/channel measurements in experimental models Indirect genetic and metabolomic evidence
Other optic neuropathies (DOA/LHON)	OPA1-mutant patient-derived iPSC RGCs PRICKLE3/MT-ND4 disease models Clinical MT-ATP6 cases	Increased reliance on reverse CX-V activity in OPA1-associated DOA Impaired CX-V assembly/stability/activity through PRICKLE3–ATP8 interactions Pathogenic MT-ATP6 variants	Reduced ΔΨm and ATP production Increased mPTP susceptibility Impaired CX-V assembly and activity	CX-V dysfunction may impair maintenance of ΔΨm and ATP production, increasing mitochondrial stress and susceptibility of RGCs to degeneration	Mixed: Direct CX-V assembly/activity measurements in some models Reverse activity inferred from oligomycin-dependent ΔΨm responses Clinical variant evidence is indirect

## Data Availability

No new data were created or analyzed in this study. Data sharing is not applicable to this article.

## Abbreviations

The following abbreviations are used in this manuscript:

Aβ	Beta-amyloid
AD	Alzheimer’s Disease
ADP	Adenosine Diphosphate
ALS	Amyotrophic Lateral Sclerosis
ATP	Adenosine Triphosphate
C9ORF72	Chromosome 9 Open Reading Frame 72
CHCHD2	Coiled-Coil-Helix-Coiled-Coil-Helix Domain-Containing Protein 2
Cryo-EM	Cryo-Electron Microscopy
CX-V	Complex V or ATP Synthase
DOA	Dominant Optic Atrophy
ETC	Electron Transport Chain
ET-1	Endothelin-1
FAD	Familial Alzheimer’s Disease
FTD	Frontotemporal Dementia
HD	Huntington’s Disease
iPSC	Induced Pluripotent Stem Cell
LHON	Leber’s Hereditary Optic Neuropathy
mPTP	Mitochondrial Permeability Transition Pore
O_2_	Oxygen
OSCP	Oligomycin Sensitivity-Conferring Protein
OXPHOS	Oxidative Phosphorylation
PD	Parkinson’s Disease
Pi	Inorganic Phosphate
PMF	Proton Motive Force
RGC	Retinal Ganglion Cells
ROS	Reactive Oxygen Species
SNCA	Synuclein Alpha Gene
ΔpH	Proton Concentration Gradient
ΔΨ	Membrane Potential
ΔΨm	Mitochondrial Membrane Potential
